# Association between living environmental quality and risk of arthritis in middle-aged and older adults: a national study in China

**DOI:** 10.3389/fpubh.2023.1181625

**Published:** 2023-06-16

**Authors:** Ri Liu, Yuefei Zhou, Yang Liu, Run Guo, Lishu Gao

**Affiliations:** ^1^Department of Orthopedics, The Second Hospital of Tangshan, Tangshan, Hebei, China; ^2^Department of Orthopedics, The First Hospital of China Medical University, Shenyang, China; ^3^Department of Biostatistics and Epidemiology, School of Public Health, China Medical University, Shenyang, China; ^4^Department of General Practice, Beijing Friendship Hospital of Capital Medical University, Beijing, China; ^5^Department of Endocrinology, Tangshan People’s Hospital, Tangshan, Hebei, China

**Keywords:** living environment quality, arthritis, cross-sectional study, cohort study, incidence

## Abstract

**Background:**

The association between combined environmental factors and the risk of arthritis is still scarcely studied. The present study performed cross-sectional and cohort studies to explore the association between risk score of living environment quality and the risk of arthritis in middle-aged and older adults in China.

**Methods:**

The study was based on China Health and Retirement Longitudinal Study (CHARLS), and it recruited 17,218 participants in the cross-sectional study and 11,242 participants in the seven-year follow-up study. The living environment quality was measured by household fuel types, household water sources, room temperature, residence types, and ambient concentration of PM2.5. Logistic regression and Cox proportional hazard regression models were utilized to examine the association between the living environment quality and the risk of arthritis. Competing risk models and stratified analyses were applied to further verify our results.

**Results:**

Compared with individuals in the suitable environment group, people who lived in moderate (OR:1.28, 95%CI: 1.14–1.43) and unfavorable environments (OR:1.49, 95%CI:1.31–1.70) showed higher risks of arthritis when considering the multiple living environmental factors (P for trend <0.001) in the cross-sectional analysis. In the follow-up study, similar results (P for trend = 0.021), moderate environment group (HR:1.26, 95%CI:1.01–1.56) and unfavorable environment group (HR: 1.36, 95%CI: 1.07–1.74), were founded.

**Conclusion:**

Inferior living environment might promote the development of arthritis. It is necessary for the public, especially old people, to improve the living environment, which may be the key to the primary prevention of arthritis.

## 1. Introduction

Arthritis is one of the most common chronic diseases and often presents with joint pain, immobility, and even joint conformity, people who suffer from arthritis may feel weakness, low self-efficacy, and social isolation, people who suffer from arthritis may feel weakness, low self-efficacy, and social isolation (1, 2). The two most common types are osteoarthritis and rheumatoid arthritis (RA). There are 355 million people with arthritis worldwide, of which 190 million have osteoarthritis, and over 16.5 million have rheumatoid arthritis (3, 4). As a leading global burden of disease, it was reported that arthritis caused a significant financial and healthcare burden in the United States in 2013 (5). According to the statistics, there are now over 100 million arthritis patients in China, half of the population aged 50 or above in China suffer from osteoarthritis and the number is increasing steadily (6, 7). In this social circumstance, it is very important to explore the risk factors of arthritis, which can be beneficial to the prevention of arthritis.

The origins and pathological causes of arthritis are complex and multifactorial, with only a limited number of factors identified as playing significant roles in its occurrence and development, including genetics and environmental factors (8–10). There were major genetic associations with the HLA locus, while multiple non-HLA genetic variants showed a relatively lower risk of RA (9). Air pollutants, including PM_2.5_ and PM_10_, have been linked to the incidence of arthritis (11, 12), and recent studies suggest that household solid fuel may pose an even greater risk. Additionally, household water sources have been implicated in the incidence of osteoarthritis (13). While living building environments, including factors such as building type, temperature, conditions, and humidity, have been studied less extensively in relation to arthritis, they can still have negative impacts on human health (14). Building type, in particular, has been associated with arthritis and is often viewed as a reflection of social and economic status (15). However, a comprehensive measurement of living quality is still uncovered.

Nowadays, most studies only paid concern to the effect of a single environmental factor that partially represents the real-living environment on arthritis. Those environmental factors could be interrelated and might have offset effects and synergistic effects on arthritis. But no related studies focused on this complex effect of multiple environmental factors on arthritis. Recently, combining multiple environmental factors, an overall quality score of the living environment was reported to have the potential to evaluate the quality of human living quality comprehensively (16). To mimic real living conditions, we included household fuel types, household water sources, room temperature, building types, and ambient concentrations of PM_2.5_ in the living environment factor score, and further assessed its effect on arthritis. All data were based on the China Health and Retirement Longitudinal Study.

## 2. Methods and materials

### 2.1. Study population

**Figure 1:**
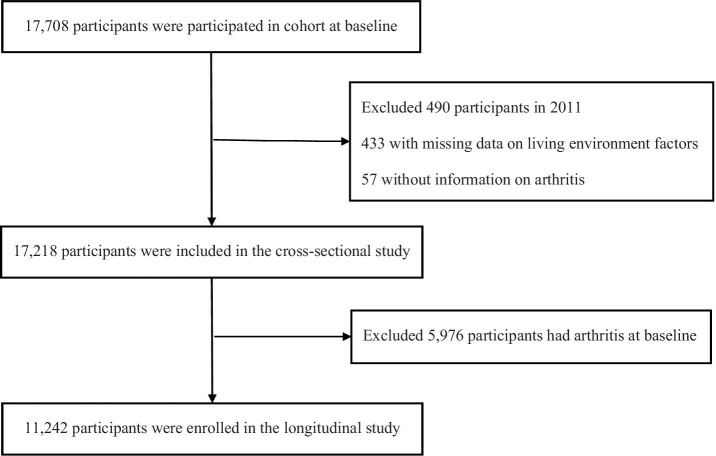
Flow diagram for participants enrolled in the study.

CHARLS is an ongoing national cohort study that recruited 17,708 participants who were successfully investigated from more than 10,000 households in 2011, the profile has been fully described elsewhere (17). Each follow-up was biennially conducted, which mainly included assisting face-to-face interviews assisted by a computer-assisted personal interviewing (CAPI) system. In the current study, we analyzed the available data from 2011 to 2018 and designed cross-sectional and longitudinal studies to detailly tap into the association between living quality and arthritis. We excluded 433 participants who had missing data on living environment factors and 57 failed to complete the information on arthritis. Thus, 17,218 participants were available in the cross-sectional study. In the longitudinal study, 11,242 participants were included after excluding 5,976 arthritics patients who had arthritis at baseline (Figure 1). This study adhered to the guidelines of the Declaration of Helsinki, and the protocol was approved by the Ethical Review Committee of Peking University (approval number: IRB 00001052-11015), and informed consent was provided by each participant.

### 2.2. Assessment of living environmental quality

The living environmental quality was mainly defined from five aspects, including atmospheric particulate concentration, domestic fuel types, household water sources, type of accommodation, and room temperature, which had been fully examined (16). The National Aeronautics and Space Administration Earth Observing System Distributed Information System published the annual average values of city-level PM_2.5_. To be specific, both the Goddard Earth Observing System chemical transport model and the geographically weighted regression model were conducted to calculate the ambient concentrations of PM_2.5_, according to the aerosol optical depth data extracted from multiple satellites (18). According to the guideline, which was issued by the Ministry of Ecology and Environment of China, 35 μg/m^3^ was determined as a cut-off value for a high level of PM_2.5_. Household fuel use for cooking and heating is the main source of indoor air pollution (19), the fuel types were further divided into two groups: clean (natural gas, marsh gas, liquefied petroleum gas, and electric for cooking; natural gas, liquefied petroleum gas, solar energy, electric, and municipal heat for heating), or solid fuels (coal, crop residue, wood, and solid charcoal for cooking; crop residue, coal, wood, and solid charcoal for heating). Furthermore, the information on building types and household water sources was collected through a standardized questionnaire, which was assisted by the CAPI system. On the other hand, interviewers got permission from the house owner entranced the house and recorded the level of temperature on the personal computer. Five levels were recorded including very hot, hot, bearable, cold, and very cold. If the temperature disturbed people, we determined it unfavorable, otherwise, suitable was implemented. The scoring method and algorithm have been detail tabulated in Table 1.

**Table 1:** The definition of living environmental quality score.

Component	Score of each component	Method of measurement
Household fuel types		Measurement: Self-reported household fuel types Examples of heating fuel measurement: “Does your residence have heating?” “What is the main heating energy source?” Examples of cooking fuel measurement: “What is the main source of cooking fuel?”
All clean fuel	0	
Mixed-fuel use	1	
All solid fuel	2	
Household water source		Measurement: Self-reported household water sources Examples tools for measurement: “Does your residence have running water?”
Tap water use	0	
Non-tap water use	1	
Room temperature		Measurement: Interviewer-observed household temperature Examples tools for measurement: [Interviewer records it] How is the temperature in this household?
Suitable	0	
Unfavorable	1	
Building type		Measurement: Self-reported building types Examples tools for measurement: “Is the building one story or multi-level building, how many stories?”
Multi-story building	0	
One-story building	1	
Ambient concentration of PM_2.5_		Extracted from the NASA Earth Observing System Distributed Information System
PM_2.5_ (<35 ug/m^3^)	0	
PM_2.5_ (≥35 ug/m^3^)	1	
Living environmental quality	Accumulation above points	The higher the score, the worse the living environmental quality

NASA, National Aeronautics and Space Administration.

### 2.3. Assessment of arthritis

The diagnosis of arthritis relies on a multi-assessment, including various clinical symptoms and imagelogical examination, and is often evaluated by a physician. In the current study, due to the data limitations, the diagnosis of arthritis was mainly based on a previous study (20). During the baseline survey, participants were asked if they had received a clinical diagnosis of arthritis from a physician. If the response was affirmative, the interviewer inquired about the onset time and recorded the information. During follow-up surveys, participants were asked to confirm the accuracy of their previous responses, and the interviewer then asked whether they had been diagnosed with arthritis since the last survey. If a patient was confirmed, the diagnostic time was recorded.

### 2.4. Assessment of covariates

The basic information of individuals on age, gender (male, female), residence (urban, rural), living with a spouse (yes, no), participating in social activity (active, inactive), annual household income (<10,000, 10,000–20,000, >20,000), education levels (illiterate, elementary school, middle school, high school or above), smoking (never, ever, current), drinking status (never, abstainer, current drinker) was collected by trained interviewers with a structured questionnaire. The category of the province was confirmed by the location of each participant. Medically trained personnel from the Chinese Center for Disease Control and Prevention collected and stored venous blood samples at −80°C. Capital Medical University’s Youanmen Center for Clinical Laboratory measured glucose (20). And they also performed the physical emanation for participants. Body mass index was calculated by a formula: BMI = weight(kg)/height^2^(m^2^), and divided into three groups according to the numerical value [normal: 18.5–24.0; thin: <18.5; overweight: ≥24.0 (21)]. An automated electronic device (OMRON Model HEM-7112, Omron Company) was used to measure the diastolic and systolic blood pressures three times on all participants’ left arms with an interval. The average of three blood pressures was calculated for the analysis. Individuals were defined as having hypertension if they self-reported physician-diagnosed hypertension, and /or their systolic blood pressures were more than 140 mmHg, and/or the diastolic blood pressures exceeded 90 mmHg (22). The levels of blood glucose were measured by the glucose oxidase method. Diabetes was evaluated by postprandial plasma glucose of more than 200 mg/dL, and/or fasting plasma glucose of at least 126 mg/dL, and/or self-reporting physician-diagnosed diabetes (23).

### 2.5. Statistical analysis

The participants were allocated into three groups which were defined according to the tertiles of living environment quality score. Means ± standard deviations (SDs) were used to describe the continuous variables and the numbers (percentages) were used to display the categorical variables. The differences in the baseline information were compared by an analysis of covariance or a chi-square test as appropriate. To examine the association between living environmental quality and arthritis risk in these two studies, we conducted two kinds of models. The logistic regression models were performed to evaluate odd ratios (ORs) with 95% confidence intervals (CIs) in the cross-sectional. While for the longitudinal study, Cox proportional hazards models with age as the time scale were established to evaluate hazard ratios (HRs) with 95% confidence intervals (CIs). Moreover, we verified whether the Cox proportional hazards model with Schoenfeld residuals met the proportional hazard assumption before establishing these models. In addition, numeric values were assigned to the tertiles of living environmental quality score and then analyzed as a continuous variable in all models to observe the trend risk between living quality and arthritis.

Stratified and interaction analyses were conducted to confirm such associations based on sex, residence, marital status, participating in social activity, hypertension status, diabetes status, annual household income, education level, body mass index, smoking status, and drinking status. Furthermore, to account for the competitive risk of death on the association between living environment and arthritis incidence, the fine and gray model was performed to reanalyze the primary results. Considering the importance of the latest WHO recommendations of PM_2.5_, we reconstructed the living environment score and re-run the statistical analysis (24). In addition, we further examined the association between each factor of living environment factors, and then summarize the weighted effect size (25) as the new living environment quality score to re-analyses the primary results. In addition, the missing data were imputed using multiple imputations, using 5 replications and chained equations in R Multiple imputations (MI). In 1–3 models, the suitable environmental group was regarded as the reference group, and all the potential confounding (age, sex, residence, marital status, education level, annual household income, BMI, participating in social activity, hypertension, diabetes, and province) were gradually fully adjusted.

All analyses were performed by using Stata version 17.0 (Stata Corp, Texas, United States) and R version 3.6.2 (R Core Team, R foundation for statistical Computing, Vienna, Austria). A two-sided *p*-value <0.05 was defined as statistical significance in all analyses.

## 3. Results

### 3.1. The baseline characteristics of study participants

The baseline characteristics of the study population are shown in Table 2 and Supplementary Table S1. A total of 17,218 individuals averagely aged 59.06 (10.14) years were included in the cross-sectional study, and 5,976 participants of them had arthritis. During an average of 7 years of follow-up, 982 new-onset arthritis were documented among 11,242 participants, which indicated an incidence rate of 8.73%. Compared with people living in suitable environments, the participants living in a worse environment were more likely to have low socioeconomic status, be isolated from society and live in rural. Meanwhile, people living in worse environments had a heavy burden of chronic diseases.

**Table 2:** Baseline characteristics of participants.

Characteristic	Total	Living environmental quality			*p*-value
		Suitable (0–1)	Moderate (2–3)	Unfavorable (4–6)	
*N*	17,218	4,323	6,342	6,553	
Age (years)	59.06 ± 10.14	58.28 ± 10.15	58.62 ± 9.99	59.99 ± 10.20	<0.001
Female, *n* (%)	8,972 (52.11)	2,292 (53.02)	3,307 (52.14)	3,373 (51.47)	0.286
Rural, *n* (%)	13,196 (76.64)	1,747 (40.41)	5,234 (82.53)	6,215 (94.84)	<0.001
Live with spouse, *n* (%)	13,813 (80.22)	3,534 (81.75)	5,051 (79.64)	5,228 (79.78)	0.014
Participating in social activity, *n* (%)	8,521 (49.49)	2,603 (60.21)	3,067 (48.36)	2,851 (43.51)	<0.001
Hypertension, *n* (%)	6,445 (37.43)	1,573 (36.39)	2,319 (36.57)	2,553 (38.96)	0.005
Diabetes, *n* (%)	2,301 (13.36)	584 (13.51)	804 (12.68)	913 (13.93)	0.106
Arthritis, *n* (%)	5,976 (34.71)	1,164 (26.92)	2,285 (36.03)	2,527 (38.56)	<0.001
Annual household income (¥), *n* (%)					<0.001
<10,000	9,849 (57.20)	2,472 (57.18)	3,491 (55.05)	3,886 (59.30)	
10,000–20,000	2,611 (15.16)	532 (12.31)	998 (15.74)	1,081 (16.50)	
>20,000	4,758 (27.64)	1,319 (30.51)	1,853 (29.22)	1,586 (24.20)	
Education level, *n* (%)					<0.001
Illiterate	7,770 (45.13)	1,256 (29.05)	2,979 (46.97)	3,535 (53.94)	
Elementary school	3,720 (21.61)	838 (19.38)	1,441 (22.72)	1,441 (21.99)	
Middle school	3,562 (20.70)	1,093 (25.28)	1,295 (20.42)	1,174 (17.92)	
High school or above	2,166 (12.58)	1,136 (26.28)	627 (9.89)	403 (6.15)	
Body mass index (kg/m^2^), *n* (%)					<0.001
Normal	8,511 (49.43)	1914 (44.27)	3,235 (51.01)	3,362 (51.30)	
Thin	1,473 (8.56)	213 (4.93)	562 (8.86)	698 (10.65)	
Overweight	7,234 (42.01)	2,196 (50.80)	2,545 (40.13)	2,404 (38.04)	
Smoking status, *n* (%)					<0.001
Never	10,415 (60.49)	2,806 (64.91)	3,855 (60.79)	3,754 (57.29)	
Ever smoker	2,020 (11.73)	502 (11.61)	717 (11.31)	801 (12.22)	
Current smoker	4,783 (27.78)	1,015 (23.48)	1,770 (27.91)	1,998 (30.49)	
Drinking status, *n* (%)					0.468
Never	10,146 (58.94)	2,584 (59.79)	3,751 (59.15)	3,811 (58.16)	
Abstainer	1,421 (8.25)	349 (8.06)	510 (8.03)	562 (8.58)	
Current drinker	5,651 (32.81)	1,390 (32.15)	2,081 (32.81)	2,180 (33.27)	
Household fuel types, *n* (%)					<0.001
All clean fuel	5,567 (32.33)	3,956 (91.51)	1,543 (24.33)	68 (1.04)	
Mixed-use of clean and solid fuel	4,618 (26.82)	367 (8.49)	2,964 (46.74)	1,287 (19.64)	
All solid fuel	7,033 (40.85)	0 (0.00)	1,835 (28.93)	5,198 (79.32)	
Non-tap water, *n* (%)	6,495 (37.72)	101 (2.34)	1,683 (26.54)	4,711 (71.89)	<0.001
Unfavorable room temperature, *n* (%)	2,841 (16.50)	178 (4.12)	839 (13.23)	1,824 (27.83)	<0.001
One-story building, *n* (%)	10,465 (60.78)	468 (10.83)	4,014 (63.29)	5,983 (91.30)	<0.001
Ambient PM_2.5_ ≥ 35 ug/m^3^, *n* (%)	9,068 (52.67)	1,586 (36.69)	3,124 (49.26)	4,358 (66.50)	<0.001

Values were means ± SD or *n* (percentages) or median (%).

Values of polytomous variables may not sum to 100% due to rounding.

### 3.2. Associations between the living environmental quality score and arthritis in the cross-sectional study

Table 3 indicated that the prevalence of arthritis was 34.66% (5,967/17,218) in total populations, and 26.92% (1,164/4,323), 36.03% (2,285/6,342), and 38.56% (2,527/6,553) were, respectively, corresponding to the suitable environmental group, moderate environmental group, and unfavorable environmental group. In the trend analyses, we demonstrated that a positive link between arthritis risk and the living environmental quality score (OR: 1.21, 95%CI: 1.14–1.29) does exist after controlling for potential confounders, which means higher arthritis risk if people lived in worsen environmental quality (*P* for trend <0.005). In the univariate analysis, people living in a moderate environment (OR:1.53, 95%CI: 1.40–1.67) and unfavorable environment (OR:1.71, 95%CI: 1.57–1.86) were more likely to have arthritis compared to those living in a suitable environment. After adjusting all covariates such as demographic factors, socioeconomic information and health-related variates in the current study, relative to the participants living in a suitable environment, individuals living in moderate (OR:1.28, 95%CI: 1.14–1.43) and unfavorable environments (OR:1.49, 95%CI: 1.31–1.70) was still associated with a higher risk of arthritis (All *P* for trend<0.05.). In the stratified and interaction analyses, though the education level and drink status modified the association between the living environmental score and arthritis, the main effects were not materially changed (Supplementary Table S7).

**Table 3:** Cross-sectional association between living environmental quality score and risk of arthritis in different models.

Living environmental quality	Number of arthritis	Prevalence	OR (95% CI)			
			Crude model^*^	Model I^†^	Model II^‡^	Model III^§^
Continuous quality score	5,976	34.66%	**1.28 (1.23, 1.34)**	**1.17 (1.11, 1.22)**	**1.14 (1.08, 1.20)**	**1.21 (1.14, 1.29)**
**Categorized quality score**						
Suitable	1,164	26.92%	1.0 (reference)	1.0 (reference)	1.0 (reference)	1.0 (reference)
Moderate	2,285	36.03%	**1.53 (1.40, 1.67)**	**1.32 (1.20, 1.45)**	**1.31 (1.18, 1.45)**	**1.28 (1.14, 1.43)**
Unfavorable	2,527	38.56%	**1.71 (1.57, 1.86)**	**1.41 (1.28, 1.56)**	**1.35 (1.21, 1.50)**	**1.49 (1.31, 1.70)**
*P*-trend			**<0.001**	**<0.001**	**<0.001**	**<0.001**

OR, odd ratio; CI, confidence interval; BMI, body mass index.

* Adjust for None.

† Adjust for Age (years), Gender (Male, Female), Residence (Urban, Rural), Marital status (Live without a spouse, Live with a spouse), Education level (Illiterate, Elementary school, Middle school, High school or above), and Annual household income (<10,000, 10,000–20,000, >20,000).

‡ Further adjust for BMI (Normal, Thin, Overweight), Smoking status (Never, Ever, Current), Drinking status (Never, Abstainer, Current), Participating in social activity (No, Yes), Hypertension (No, Yes), and Diabetes (No, Yes).

§ Additionally adjusted for Province (Categorized by name of each province).

### 3.3. Associations between the living environmental risk score and arthritis at follow-up 2011–2018

Table 4 shows that during a median of seven-years follow-up, 982 arthritis was recorded. The incidence per 1,000 person-years was also calculated in each group. The incidence of arthritis was 13.56 per 1,000 person-years in the entire cohort. The incidences of arthritis were 9.95, 14.35, and 15.69 per 1,000 person-years in suitable, moderate, and unfavorable environment groups when the score was a categorized variable. The results of the cohort study were basically consistent with the cross-sectional study. In the trend analyses, we found that a positive link between arthritis risk and the living environmental quality score (HR: 1.14, 95%CI: 1.02–1.29) does exist after controlling for potential confounders, which means higher arthritis risk if people lived in worsen environmental quality (*P* for trend <0.005). When dividing the participants into different groups, the crude and full-adjusted models also showed monotonous increasing trends between arthritis risk and poor living environment (All *P* for trend <0.05). Individuals living in moderate (HR:1.26, 95%CI: 1.01–1.56) and unfavorable (HR: 1.36, 95%CI: 1.07–1.74) environments met an increased risk of arthritis after adjusting for all potential confounders, compared to the suitable environment group. As shown in Supplementary Table S8, no effect modifier was detected (all *P* for interaction >0.05) and the results in each stratum largely sided with the primary results, which further examined the stability of our findings. Besides, after considering the competition of deaths in the cohort study, the results in Fine & Grey models were still stable and similar to the major results (Supplementary Table S2). The results are consistent with primary results after weighting effect size as the new living environment quality score and the results were still kept in line with primary results after MI (Supplementary Tables S3, S4). The results are consistent with the primary results after utilizing the WHO recommendations as cutoff points for PM2.5 (Supplementary Tables S5, S6).

**Table 4:** Longitudinal association between living environmental quality score and arthritis in different models.

Living environmental quality	Number of arthritis	Incidence rate per 1,000 person-years	HR (95% CI)			
			Crude model^*^	Model I^†^	Model II^‡^	Model III^§^
Continuous quality score	982	13.56	**1.23 (1.14, 1.33)**	**1.15 (1.05, 1.26)**	**1.12 (1.02, 1.24)**	**1.14 (1.02, 1.29)**
**Categorized quality score**						
Suitable	207	9.95	1.0 (reference)	1.0 (reference)	1.0 (reference)	1.0 (reference)
Moderate	376	14.35	**1.44 (1.22, 1.71)**	**1.30 (1.08, 1.57)**	**1.30 (1.07, 1.59)**	**1.26 (1.01, 1.56)**
Unfavorable	399	15.69	**1.57 (1.33, 1.86)**	**1.38 (1.13, 1.67)**	**1.33 (1.08, 1.63)**	**1.36 (1.07, 1.74)**
*P*-trend			**<0.001**	**0.003**	**0.018**	**0.021**

HR, hazards ratio; CI, confidence interval.

* Adjust for Age as time scale.

† Adjust for Age as time scale, Gender (Male, Female), Residence (Urban, Rural), Marital status (Live without spouse, Live with spouse), Education level (Illiterate, Elementary school, Middle school, High school or above) and Annual household income (<10,000, 10,000–20,000, >20,000).

‡ Further adjust for BMI (Normal, Thin, Overweight), Smoking status (Never, Ever, Current), Drinking status (Never, Abstainer, Current), Participating in social activity (No, Yes), Hypertension (No, Yes), and Diabetes (No, Yes).

§ Additionally adjusted for Province (Categorized by name of each province).

## 4. Discussion

In the present study, both cross-sectional and longitudinal studies found poorer living environmental quality associated with a higher risk of arthritis. This reminds us that we need to concentrate on the overall living environmental quality, which is beneficial to older people to alleviate the heavy burden of disease.

Indoor air pollution poses a great threat to human health. A higher risk of chronic multimorbidity due to household air pollution was identified in Chinese adults (26, 27). It was reported that about 50% of individuals might choose solid fuels, such as coal, kerosene, and biomass fuels, for domestic use for cooking and heating, especially in some developing countries (28). When these solid fuels are burned, household air pollution came after. One study from WHO has indicated that household air pollution might result in about 2 million deaths per year (29). Recently a nationwide population-based cohort study showed that the incidence of arthritis was lower among clean fuel users than solid fuel users, as a concrete manifestation of comparing to clean fuel users, cooking and heating with solid fuels had a relatively higher risk of arthritis (28). Another cohort study demonstrated the same trend for cooking with solid fuel compared with cleaner fuel (20). Meanwhile, a cross-sectional study comprehensively showed that the use of gas, coal, wood, or biomass fuels for cooking was greatly associated with increased odds of arthritis, compared to electricity (30). Those evidences hinted us we need to incorporate household fuel types as one of the important elements to estimate the comprehensive living environmental quality. People consider it difficult to afford the high cost of clean fuel used domestically, especially for an economically underdeveloped country like China. Thus, they prefer cheap fuel types and cleaner fuel use may be a barrier to tackling the health burden (31). which means the process of switching from solid fuel to clean one can be chronic and indicates the household fuel types is relatively stable. Solid fuels play a leading role in heating in northern China (32). and improving income levels is not predicted to alter the pattern of mixed use of multiple fuels in China (33, 34). This evidence elucidated that unhealthy energy is a long-term lifestyle habit that often persists over a lifetime.

Water is a basic need of life and the quality of used water was identified as a leading factor involved in the pathogenesis of many diseases. A follow-up study has already indicated that individuals suffered from complaints and symptoms after household water contamination (35). Household water use was often polluted by heavy metals and many other kinds of toxic elements (36, 37). Tap water in residences is always uniformly filtered by the government so that it tends to be healthier than other water sources such as groundwater and well water. To the best of our knowledge, most studies mainly focused on the effect of water contamination and arsenic exposure on human health (38, 39). Only one study showed that drinking spring water and the well water was associated with a higher risk of osteoarthritis compared to drinking tap water. These studies indicated household water sources play an essential in people’s everyday life and impact residents’ health. However, the use of water sources was often mixed, so further studies are supposed to explore the specific mechanisms of the development of arthritis.

When climate change came into the researcher’s eyes, it was confirmed to be a major contributor to arthritis (13). The physiological functions of the human body could be directly influenced by temperature changes and the incidence of illness and mortality related to cold and heat stress might increase significantly. A meta-analysis reported that climate change may influence the clinical care and pain reporting of patients with RA (40). Likewise, one study from China reported that temperature decrease was significantly associated with RA admission (25th percentile of temperature vs. 50th percentile of temperature), with the acute and largest effect at current days lag (41). Another retrospective cohort study showed minimum temperature has promoted effect on the pain of rheumatoid arthritis (42). In contrast, fewer studies focused on the association between indoor room temperature and arthritis. An internet-based case-crossover study in the United States demonstrated that higher temperatures were related to approximately 40% higher risk of a gout attack, a chronic disease with inflammatory arthritis, compared to moderate temperatures (43). In basic research, a mouse model with Chikungunya (CHIKV) infection and arthritis showed that CHIKV replication and foot arthropathy were reduced by housing at 30°C compared to 22°C (44). In our study, the unfavorable temperature in the residence was considered a potential risk factor for arthritis. However, certain measured values for household temperature could not be obtained, so more studies were still supposed to assess the effects of indoor room temperature on arthritis.

Air pollution is a major environmental risk to public health. Almost the whole global population is exposed to air pollution which exceeds the standard of World Health Oganization (WHO) for PM_2.5_. The specific mechanisms behind air pollution-caused arthritis might include increased oxidative stress, epigenetic modifications, and systemic inflammation induced by exposures and immune response (45). Recently a case-crossover study including 888 patients with RA showed a striking association between air pollution and the severity of RA and reactivations. Higher levels of air pollutants were associated with increased C-reaction protein (CRP) levels and a higher risk of RA flare. Consistently, several studies from various countries have indicated the correlation between PM_2.5_ exposure and arthritis risk. A study including 722,885 individuals in Taiwan showed an increased risk of developing RA in exposure to PM_2.5_ (12). Meanwhile, a time-series study demonstrated that high-concentration PM_2.5_ was associated with RA remission (46). One animal study also showed a significant effect of exposure to particular matters (PMs) and PMs gaseous exposure with osteoarthritis in rats (47). However, some studies showed that air pollution might not be associated with the risk of RA. A systemic meta-analysis demonstrated that there was an inverse effect between PM_2.5_ and arthritis (48). Results from the Swedish Epidemiological Investigation of Rheumatoid Arthritis (EIRA) case–control study also showed that PM_10_ was not associated with the increased risk of RA (49). Similarly, another study from Taiwan showed, among four quantiles of PM_2.5_ concentration, demonstrated a risk trend between PM_2.5_ and RA although the results were statistically insignificant (50). In our study, higher PM_2.5_ concentration was listed as a potential risk factor in the environmental score to estimate the overall living environment and its effect on arthritis. Those results indicated a complex association between PMs and arthritis. Different types of arthritis may have different relations to exposure to PM_2.5_. For example, the correlation between rheumatology arthritis and exposure to PM_2.5_ may differ from osteoarthritis. As one recent study reported that the models with 89% weighting the late-stage (>40 years) indicated PM_2.5_ exposure was not associated with gout or osteoarthritis (51). However, the results remain controversial. Many studies indicated the detrimental effect of PM_2.5_ on overall arthritis (30, 52). Indeed, future studies need to explore the relationship between specific arthritis and PM_2.5_ exposure and identify the association between our score and specific arthritis. Any extrapolation on the association between the score and specific types of arthritis based on our study should be careful.

The environments of the living and working buildings were reported to impose an effect on human health. Whereas, few studies focused on the role of the built environments of various neighborhoods in the development of arthritis. Most concentrated on the impact of the built environment on physical activity and pain in arthritis patients. Results of the 2015 National Health Interview Survey Data showed that less than a highly-walkable neighborhood and lower social cohesion were independently associated with decreased odds of meeting physical activity recommendations among adults with arthritis and recent joint pain (53). Another scope review revealed that a neighborhood-built environment was very important for supporting osteoarthritis self-management, especially for facilitating physical activity (54). One cross-sectional study in Finland, Poland, and Spain demonstrated that the improvement of neighborhood features could facilitate the mobility of the aging population, with evidence of benefits for health (55). What’s more, people living in older households tended to have abnormal clinical features of ankylosing spondylitis, compared to people who lived in buildings that were developed in 1990 or after (56). Moreover, specific attributes of the neighborhood-built environment were greatly associated with physical activity in older people with lower limb osteoarthritis than those without it (57). The influence of the type of housing on arthritis is still obscure. A study from China has investigated the association between the environment of buildings, including types of buildings, and self-reported health status (58). That study found living in a multi-story building was a risk factor for health-related challenges compared to residing in a single-story house and high-rise elevator buildings among older persons. In the present study, it was reported that living in a single-story building, compared to living in a multi-story housing, was listed as a potential risk factor associated with a greater risk of arthritis. The discrepancy may vary across ages and differ by different outcomes. It was reported that the older population living in rural areas and staying at lower socioeconomic status was supposed to suffer from arthritis (14). This phenomenon is more common in China, multi-floor housings were very popular in urban areas since the 1950s, they were built by the government and used for nation-owned companies’ work units for their laborers to live in; however, many people from disadvantaged backgrounds still tent to live in single-story buildings at that time (59).

The environment of human living was very complex and filled with indoor and outdoor pollutants. Numerous studies have demonstrated the correlation between a single environmental factor and arthritis. However, studies about the combined effects of various environmental factors were very limited, so it was prompt to explore the integrated risk factors for arthritis in the living environment. To explore the comprehensive environmental exposure in people’s living environments, a score combining five different items in our daily life was utilized to evaluate the possible exposure level of every person, simulate to the scores of a healthy lifestyle. Through a cross-sectional and seven-year cohort study, the results of our study showed that there is a detrimental effect of poor environmental living quality on arthritis. In the cross-sectional analyses, education level and drinking status were identified to modify the association between the living environmental score and arthritis, although the trend remained the same as the main results. Education levels are straightly related to working types and environment and reflect socioeconomic status to some degree. Our results indicated that those with higher education levels living in worse environmental quality were supposed to have a higher risk of arthritis and more attention should be paid to this group. The effect of alcohol consumption on arthritis remained, and its effect on arthritis sustained although people have quit drinking, which means alcohol intake can pose a chronic impact on arthritis. Additionally, the specific alcohol intake was not measured in our study due to the data limitation. Future studies are needed to study how alcohol consumption modified the association between living environmental quality score and arthritis. Nevertheless, the specific mechanisms of how the environment influences arthritis were not studied completely. Many pathways regulated by the pollutants in different media were identified to involve in arthritis responses. For example, pollutants inhaled in the lung could trigger pro-inflammatory or oxidative stress mediators and take part in the internal circulation of the human body. Meanwhile, the imbalance of the autonomic nervous system could be produced by these pollutants. Moreover, some elements of the pollutants might straightly get into the bone and skeletal tissues and participate in the development of arthritis. These pollutants could induce subclinical physiological changes, such as synovium injury, fatigue, movement disorder, vasoconstriction and endothelial dysfunction, which might direct the actual cause of the arthritis event in a single person.

To the best of our knowledge, the present study was the first to analyze the effects of comprehensive living quality on arthritis utilizing the comprehensive score. An integrated environment, including indoor and outdoor environments, as well as chemical elements and physical factors, was estimated to provide the most accurate description of environmental exposures. The findings in our study could well illustrate the casual association according to the cross-sectional and cohort study. Above all, the burden of arthritis was one of the most serious public health problems. Our study could help to discover the high-risk populations and carry out preventive methods timely. However, the present study still has some limitations to notice. First, the diagnosis of arthritis relies on self-reported physician-diagnosed arthritis, which means the number of patients and association of living quality with arthritis may be underestimated. However the diagnostic method was validated, and the statistical results still hold (60). Second, we focused on the association between baseline exposure and arthritis but the exposure may change as time goes by. Therefore, future studies should consider measuring this association more comprehensively. Third, the weighted scores have been used in our study, the results were not changed significantly, and another study proved the validity of the current living environment score (19, 20). Nonetheless, there are still some improvement areas in the algorithm of living environment score due to many other factors also existing in people’s living settings. Fourth, although the temperature indoors is relatively steady for humans and has been used in some studies (16), the temperature outdoors is varying across different times, and further studies are needed to elucidate the association between the fluctuation of temperature and arthritis. Fifth, the data on certain types of arthritis was lacking, and the impact of our score on particular arthritis could not be validated in detail and the results may vary. So, any extrapolation about the effect of living environment quality on specific types of arthritis based on this study should be approached with caution although the association between living environment and overall arthritis has been found. And future studies are needed to explore the association between the living environmental quality score and certain types of arthritis. Finally, many confounding factors may still exist although the current study included many significant confounders including demographic factors, lifestyle, social status, and clinical features.

## 5. Conclusion

Worse living environmental quality was adversely correlated to arthritis, both in the cross-sectional and the seven-year follow-up studies. We consider it necessary to attract the attention of the public and the government to the importance of spending efforts to improve the living environment.

## Data availability statement

Publicly available datasets were analyzed in this study. This data can be found here: All the data can be obtained from the China Health and Retirement Longitudinal Study 2011 to 2018 (http://charls.pku.edu.cn/).

## Ethics statement

The studies involving human participants were reviewed and approved by the study protocol was approved by Peking University’s Ethical Review Committee (IRB0000105211015). The patients/participants provided their written informed consent to participate in this study.

## Author contributions

LG, YL, and RL: designed and devised. YZ, RL, and RG: control. YZ, RL and RG: writing and translation. YL, LG, and YZ: revising and reviewing. All authors contributed to the article and approved the submitted version.

## Funding

The study was supported by Medical Science Research Project of Hebei Province in 2020 (NO. 20201458) and Medical Science Research Project of Hebei Province in 2021 (NO. 20211157).

## Conflict of interest

The authors declare that the research was conducted in the absence of any commercial or financial relationships that could be construed as a potential conflict of interest.

## Publisher’s note

All claims expressed in this article are solely those of the authors and do not necessarily represent those of their affiliated organizations, or those of the publisher, the editors and the reviewers. Any product that may be evaluated in this article, or claim that may be made by its manufacturer, is not guaranteed or endorsed by the publisher.
