# Supplementary material for: Association between living environmental quality and risk of arthritis in middle-aged and older adults: a national study in China
Source: Front Public Health. 2023 Jun 16;11:1181625. doi: 10.3389/fpubh.2023.1181625 (PMC10313337; doi:10.3389/fpubh.2023.1181625)
Supplement: Supplementary file 1 [file Data_Sheet_1.pdf]

Supplementary Material

Association between living environmental quality and risk of arthritis in middle-aged and elderly adults: a national study in China

Ri Liu[1#], Yuefei, Zhou[2#], Yang, Liu[3], Run, Guo[4], Lishu, Gao[5]

*** Correspondence:** Lishu Gao, Department of Endocrinology, Tangshan People's Hospital, Tangshan, Hebei, 063000, China; E-mail: gaolishu2022@126.comSupplementary Data

[#]Liu Ri and Zhou Yuefei contributed equally to the article.

Table S1 Baseline characteristics of participants including missing values.
Table S2 T Longitudinal association between living environmental quality score and incident arthritis in the Fine-Gray models
Table S3 Longitudinal association between weighted living environmental quality score and incident arthritis in the cohort study.
Table S4 Longitudinal association between living environmental quality score and incident arthritis in different models after the missing data were imputed using multiple imputations.
Table S5 Cross-sectional association between living environmental quality score (using the WHO recommendations as cutoff points for PM2.5) and risk of arthritis in different models.
Table S6 Longitudinal association between living environmental quality score (using the WHO recommendations as cutoff points for PM2.5) and arthritis in different models
Table S7 Stratified and interaction analyses of between living environmental quality and risk of arthritis in cross-sectional analysis.
Table S8 Stratified and interaction analyses of between living environmental quality and risk of arthritis in longitudinal analysis.

Table S1 Baseline characteristics of participants including missing values.

Characteristic	Total	Living Environmental Quality			*P*-value
		Suitable (0-1)	Moderate (2-3)	Unfavorable (4-6)	
N	17218	4323	6342	6553	
Age (years)	59.06 ± 10.14	58.28 ± 10.15	58.62 ± 9.99	59.99 ± 10.20	<0.001
Female, n (%)	8970 (52.10)	2292 (53.02)	3306 (52.14)	3372 (51.47)	0.283
Rural, n (%)	13192 (76.64)	1747 (40.41)	5232 (82.52)	6213 (94.84)	<0.001
Live with spouse, n (%)	13813 (80.22)	3534 (81.75)	5051 (79.64)	5228 (79.78)	0.014
Participating in social activity, n (%)	7964 (50.06)	2407 (60.39)	2864 (48.96)	2693 (44.35)	<0.001
Hypertension, n (%)	6439 (37.44)	1572 (36.39)	2316 (36.58)	2551 (38.98)	0.005
Diabetes, n (%)	2294 (13.36)	581 (13.47)	803 (12.69)	910 (13.92)	0.119
Arthritis, n (%)	5967 (34.66)	1164 (26.92)	2285 (36.03)	2527(38.56)	<0.001
Annual household income (¥), n (%)					<0.001
<10000	9817 (57.24)	2454 (57.18)	3482 (55.16)	3881 (59.34)	
10000-20000	2595 (15.13)	529 (12.31)	990 (15.68)	1076 (16.45)	
>20000	4739 (27.63)	1315 (30.60)	1841 (29.16)	1583 (24.20)	
Education level, n (%)					<0.001
Illiterate	7762 (45.14)	1253 (29.08)	2976 (46.95)	3533 (53.95)	
Elementary school	3715 (21.60)	834 (19.35)	1441 (22.73)	1440 (21.99)	
Middle school	3560 (20.70)	1091 (25.32)	1295 (20.43)	1174 (17.93)	
High school or above	2160 (12.56)	1131 (26.25)	627 (9.89)	402 (6.14)	
Body mass index (kg/m^2), n (%)					<0.001
Normal	7828 (52.04)	1685 (48.45)	3002 (53.53)	3141 (52.75)	
Thin	1012 (6.73)	145 (4.17)	377 (6.72)	490 (8.23)	
Overweight	6201 (41.23)	1648 (47.38)	2229 (39.75)	2324 (39.03)	
Smoking status, n (%)					<0.001
Never	10409 (60.48)	2803 (64.90)	3854 (60.78)	3752 (57.28)	
Ever smoker	2018 (11.73)	501 (11.60)	717 (11.31)	800 (12.21)	
Current smoker	4783 (27.79)	1015 (23.50)	1770 (27.91)	1998 (30.50)	
Drinking status, n (%)					0.462
Never	10137 (58.94)	2581 (59.79)	3748 (59.15)	3808 (58.17)	
Abstainer	1419 (8.25)	348 (8.06)	509 (8.03)	562 (8.59)	
Current drinker	5643 (32.81)	1388 (32.15)	2079 (32.81)	2176 (33.24)	
Household fuel types, n (%)					<0.001
All clean fuel	5567 (32.33)	3956 (91.51)	1543 (24.33)	68 (1.04)	
Mixed-use of clean and solid fuel	4618 (26.82)	367 (8.49)	2964 (46.74)	1287 (19.64)	
All solid fuel	7033 (40.85)	0 (0.00)	1835 (28.93)	5198 (79.32)	
Non-tap water, n (%)	6495 (37.72)	101 (2.34)	1683 (26.54)	4711 (71.89)	<0.001
Unfavorable room temperature, n (%)	2841 (16.50)	178 (4.12)	839 (13.23)	1824 (27.83)	<0.001
One-story building, n (%)	10465 (60.78)	468 (10.83)	4014 (63.29)	5983 (91.30)	<0.001
Ambient $PM_{2.5} \geq 35$ ug/m^3, n (%)	9068 (52.67)	1586 (36.69)	3124 (49.26)	4358 (66.50)	<0.001

Values were means ± SD or n (percentages) or median (%).

Values of polytomous variables may not sum to 100% due to rounding.

Table S2 Longitudinal association between living environmental quality score and incident arthritis in the Fine-Gray models.

Living environmental quality	Number of arthritis	Incidence Rate per 1000 Person-Years	HR (95% CI)			
			Crude Model*	Model I†	Model II‡	Model III§
Continuous quality score	982	13.56	**1.22 (1.13, 1.32)**	**1.14 (1.05, 1.25)**	**1.12 (1.02, 1.23)**	**1.14 (1.02, 1.28)**
Categorized quality score						
Suitable	207	9.95	1.0 (Reference)	1.0 (Reference)	1.0 (Reference)	1.0 (Reference)
Moderate	376	14.35	**1.43 (1.21, 1.69)**	**1.30 (1.08, 1.56)**	**1.31 (1.08, 1.58)**	**1.25 (1.01, 1.55)**
Unfavorable	399	15.69	**1.54 (1.30, 1.82)**	**1.36 (1.13, 1.65)**	**1.32 (1.08, 1.61)**	**1.35 (1.06, 1.71)**
P-trend			**<0.001**	**0.003**	**0.018**	**0.025**

Abbreviations: HR, hazards ratio; CI, confidence interval.

*Adjust for Age as time scale;

†Adjust for Age as time scale, Gender (Male, Female), Residence (Urban, Rural), Marital status (Live without spouse, Live with spouse), Education level (Illiterate, Elementary school, Middle school, High school or above), and Annual household income (<10000, 10000-20000, >20000);

‡Further adjust for BMI (Normal, Thin, Overweight), Smoking status (Never, Ever, Current), Drinking status (Never, Abstainer, Current), Participating in social activity (No, Yes), Hypertension (No, Yes), and Diabetes (No, Yes);

§Additionally adjusted for Province (Categorized by name of each province).

Table S3 Longitudinal association between weighted living environmental quality score and incident arthritis in the cohort study.

Living environmental quality	Number of arthritis	Incidence Rate per 1000 Person-Years	HR (95% CI)			
			Crude Model*	Model I†	Model II‡	Model III§
Continuous quality score	982	13.56	**1.20 (1.11, 1.30)**	**1.13 (1.03, 1.23)**	**1.12 (1.02, 1.23)**	**1.11 (1.00, 1.23)**
Categorized quality score						
Suitable	260	10.38	1.0 (Reference)	1.0 (Reference)	1.0 (Reference)	1.0 (Reference)
Moderate	361	15.19	**1.46 (1.24, 1.71)**	**1.31(1.10, 1.56)**	**1.34 (1.11, 1.62)**	**1.34 (1.10, 1.63)**
Unfavorable	361	17.57	**1.46(1.25, 1.72)**	**1.31 (1.09, 1.57)**	**1.31 (1.08, 1.58)**	**1.28 (1.04, 1.59)**
P-trend			**<0.001**	**0.007**	**0.016**	**0.050**

Abbreviations: HR, hazards ratio; CI, confidence interval.

*Adjust for Age as time scale;

†Adjust for Age as time scale, Gender (Male, Female), Residence (Urban, Rural), Marital status (Live without spouse, Live with spouse), Education level (Illiterate, Elementary school, Middle school, High school or above), and Annual household income (<10000, 10000-20000, >20000);

‡Further adjust for BMI (Normal, Thin, Overweight), Smoking status (Never, Ever, Current), Drinking status (Never, Abstainer, Current), Participating in social activity (No, Yes), Hypertension (No, Yes), and Diabetes (No, Yes);

§Additionally adjusted for Province (Categorized by name of each province).

Table S4 Longitudinal association between living environmental quality score and incident arthritis in different models after the missing data were imputed using multiple imputations.

Living environmental quality	Number of arthritis	Incidence Rate per 1000 Person-Years	HR (95% CI)			
			Crude Model*	Model I†	Model II‡	Model III§
Continuous quality score	982	13.56	**1.23 (1.14, 1.33)**	**1.14 (1.04, 1.24)**	**1.13 (1.04, 1.24)**	**1.14 (1.04, 1.25)**
Categorized quality score						
Suitable	207	9.95	1.0 (Reference)	1.0 (Reference)	1.0 (Reference)	1.0 (Reference)
Moderate	376	14.35	**1.44 (1.22, 1.71)**	**1.29 (1.07, 1.55)**	**1.29 (1.07, 1.55)**	**1.28 (1.06, 1.54)**
Unfavorable	399	15.69	**1.57 (1.33, 1.85)**	**1.36 (1.12, 1.65)**	**1.35 (1.11, 1.64)**	**1.36 (1.12, 1.65)**
P-trend			**<0.001**	**0.007**	**0.007**	**0.005**

Abbreviations: HR, hazards ratio; CI, confidence interval.

*Adjust for Age as time scale;

†Adjust for Age as time scale, Gender (Male, Female), Residence (Urban, Rural), Marital status (Live without spouse, Live with spouse), Education level (Illiterate, Elementary school, Middle school, High school or above) and Annual household income (<10000, 10000-20000, >20000);

‡Further adjust for BMI (Normal, Thin, Overweight), Smoking status (Never, Ever, Current), Drinking status (Never, Abstainer, Current), Participating in social activity (No, Yes), Hypertension (No, Yes), and Diabetes (No, Yes);

§Additionally adjusted for Province (Categorized by name of each province).

Table S5 Cross-sectional association between living environmental quality score (using the WHO recommendations as cutoff points for $PM_{2.5}$) and risk of arthritis in different models.

Living environmental quality	Number of arthritis	Prevalence	OR (95% CI)			
			Crude Model*	Model I†	Model II‡	Model III§
Continuous quality score	5976	34.66%	**1.37 (1.31, 1.43)**	**1.25 (1.19, 1.31)**	**1.21 (1.15, 1.28)**	**1.23 (1.15, 1.31)**
Categorized quality score						
Suitable	1073	17.96%	1.0 (Reference)	1.0 (Reference)	1.0 (Reference)	1.0 (Reference)
Moderate	1756	29.38%	**1.48 (1.34, 1.63)**	**1.27 (1.14, 1.42)**	**1.30 (1.15, 1.46)**	**1.28 (1.13, 1.47)**
Unfavorable	3147	52.66%	**1.92 (1.75, 2.11)**	**1.57 (1.40, 1.76)**	**1.51 (1.34, 1.71)**	**1.54 (1.34, 1.77)**
P-trend			**<0.001**	**<0.001**	**<0.001**	**<0.001**

Abbreviations: OR, odd ratio; CI, confidence interval; BMI, body mass index.

*Adjust for None;

†Adjust for Age (years), Gender (Male, Female), Residence (Urban, Rural), Marital status (Live without a spouse, Live with a spouse), Education level (Illiterate, Elementary school, Middle school, High school or above), and Annual household income (<10000, 10000-20000, >20000);

‡Further adjust for BMI (Normal, Thin, Overweight), Smoking status (Never, Ever, Current), Drinking status (Never, Abstainer, Current), Participating in social activity (No, Yes), Hypertension (No, Yes), and Diabetes (No, Yes);

§Additionally adjusted for Province (Categorized by name of each province).

Table S6 Longitudinal association between living environmental quality score (using the WHO recommendations as cutoff points for $PM_{2.5}$) and arthritis in different models

Living environmental quality	Number of arthritis	Incidence Rate per 1000 Person-Years	HR (95% CI)			
			Crude Model*	Model I†	Model II‡	Model III§
Continuous quality score	982	13.56	**1.26 (1.15, 1.37)**	**1.17 (1.06, 1.29)**	**1.16 (1.04, 1.29)**	**1.15 (1.02, 1.29)**
Categorized quality score						
Suitable	148	6.15	1.0 (Reference)	1.0 (Reference)	1.0 (Reference)	1.0 (Reference)
Moderate	328	13.63	**1.46 (1.20, 1.77)**	**1.31 (1.05, 1.62)**	**1.33 (1.05, 1.68)**	**1.29 (1.01, 1.64)**
Unfavorable	506	15.57	**1.66 (1.38, 2.00)**	**1.45 (1.16, 1.80)**	**1.43 (1.13, 1.81)**	**1.38 (1.07, 1.79)**
P-trend			**<0.001**	**0.001**	**0.006**	**0.027**

Abbreviations: HR, hazards ratio; CI, confidence interval.
*Adjust for Age as time scale;
†Adjust for Age as time scale, Gender (Male, Female), Residence (Urban, Rural), Marital status (Live without spouse, Live with spouse), Education level (Illiterate, Elementary school, Middle school, High school or above) and Annual household income (<10000, 10000-20000, >20000);
‡Further adjust for BMI (Normal, Thin, Overweight), Smoking status (Never, Ever, Current), Drinking status (Never, Abstainer, Current), Participating in social activity (No, Yes), Hypertension (No, Yes), and Diabetes (No, Yes);
§Additionally adjusted for Province (Categorized by name of each province).

Table S7 Stratified and interaction analyses of between living environmental quality and risk of arthritis in cross-sectional analysis

Characteristics	Living Environmental Quality, OR (95% CI) †				*P*-trend	*P*-interaction
	Continuous variable	Suitable (0-1)	Moderate (2-3)	Unfavorable (4-6)		
Age (years)						0.765
≤60 (N=10341)	1.21 (1.11, 1.31)	1.0 (Reference)	1.32 (1.14, 1.53)	1.50 (1.26, 1.77)	<0.001	
>60 (N=6869)	1.21 (1.10, 1.34)	1.0 (Reference)	1.18 (0.98, 1.42)	1.45 (1.18, 1.79)	<0.001	
Gender						0.122
Men (N=8246)	1.21 (1.10, 1.33)	1.0 (Reference)	1.27 (1.06, 1.51)	1.49 (1.22, 1.81)	<0.001	
Women (N=8970)	1.22 (1.12, 1.32)	1.0 (Reference)	1.30 (1.12, 1.52)	1.52 (1.28, 1.81)	<0.001	
Residence						0.144
Urban (N=4022)	1.24 (1.06, 1.44)	1.0 (Reference)	1.43 (1.16, 1.77)	1.36 (0.97, 1.90)	0.006	
Rural (N=13192)	1.20 (1.12, 1.28)	1.0 (Reference)	1.19 (1.03, 1.37)	1.42 (1.22, 1.66)	<0.001	
Marital status						0.053
Live without spouse (N=3405)	1.16 (1.00, 1.34)	1.0 (Reference)	1.26 (0.96, 1.66)	1.38 (1.02, 1.88)	0.050	
Live with spouse (N=13813)	1.22 (1.14, 1.31)	1.0 (Reference)	1.29 (1.14, 1.46)	1.52 (1.32, 1.76)	<0.001	
Participating in social activity						0.856
Inactive (N=7944)	1.22 (1.12, 1.33)	1.0 (Reference)	1.23 (1.04, 1.46)	1.50 (1.25, 1.80)	<0.001	
Active (N=7964)	1.20 (1.10, 1.31)	1.0 (Reference)	1.32 (1.12, 1.55)	1.48 (1.23, 1.78)	<0.001	
Hypertension						0.351
No (N=10757)	1.17 (1.08, 1.27)	1.0 (Reference)	1.17 (1.01, 1.36)	1.37 (1.16, 1.62)	<0.001	
Yes (N=6439)	1.28 (1.16, 1.41)	1.0 (Reference)	1.46 (1.21, 1.75)	1.72 (1.39, 2.11)	<0.001	
Diabetes						0.861
No (N=14883)	1.20 (1.12, 1.28)	1.0 (Reference)	1.25 (1.10, 1.41)	1.46 (1.26, 1.67)	<0.001	
Yes (N=2294)	1.29 (1.09, 1.53)	1.0 (Reference)	1.51 (1.10, 2.07)	1.76 (1.24, 2.51)	0.003	
Annual household income (¥)						0.848
<10000 (N=9817)	1.22 (1.12, 1.33)	1.0 (Reference)	1.24 (1.06, 1.45)	1.50 (1.26, 1.78)	<0.001	
10000-20000 (N=2595)	1.26 (1.07, 1.48)	1.0 (Reference)	1.29 (0.95, 1.74)	1.61 (1.14, 2.26)	0.005	
>20000 (N=4739)	1.16 (1.03, 1.30)	1.0 (Reference)	1.42 (1.15, 1.76)	1.43 (1.12, 1.82)	0.015	
Education level						0.002
Illiterate (N=7762)	1.21 (1.11, 1.33)	1.0 (Reference)	1.19 (1.00, 1.42)	1.46 (1.21, 1.77)	<0.001	
Elementary school (N=3715)	1.10 (0.96, 1.25)	1.0 (Reference)	1.18 (0.92, 1.50)	1.24 (0.94, 1.63)	0.157	
Middle school (N=3560)	1.24 (1.08, 1.43)	1.0 (Reference)	1.25 (0.97, 1.60)	1.55 (1.16, 2.08)	0.003	
High school or above (N=2160)	1.56 (1.25, 1.95)	1.0 (Reference)	1.97 (1.39, 2.79)	2.45 (1.57, 3.83)	<0.001	
Body mass index (kg/m^2)						0.188
Normal (N=7828)	1.17 (1.07, 1.27)	1.0 (Reference)	1.32 (1.12, 1.55)	1.43 (1.20, 1.72)	<0.001	
Thin (N=1012)	1.16 (0.90, 1.49)	1.0 (Reference)	0.79 (0.47, 1.31)	1.11 (0.65, 1.91)	0.258	
Overweight (N=6201)	1.28 (1.16, 1.41)	1.0 (Reference)	1.32 (1.10, 1.57)	1.65 (1.35, 2.03)	<0.001	
Smoking status						0.280
Never (N=10409)	1.24 (1.14, 1.34)	1.0 (Reference)	1.33 (1.15, 1.53)	1.57 (1.33, 1.84)	<0.001	
Ever smoker (N=2018)	1.33 (1.07, 1.66)	1.0 (Reference)	1.44 (0.96, 2.17)	1.82 (1.15, 2.89)	0.011	
Current smoker (N=4783)	1.13 (1.00, 1.27)	1.0 (Reference)	1.14 (0.91, 1.43)	1.28 (0.99, 1.64)	0.046	
Drinking status						0.012
Never (N=10137)	1.18 (1.09, 1.28)	1.0 (Reference)	1.20 (1.04, 1.39)	1.40 (1.18, 1.65)	<0.001	
Abstainer (N=1419)	1.27 (1.02, 1.59)	1.0 (Reference)	1.53 (1.00, 2.35)	1.75 (1.09, 2.81)	0.033	
Current drinker (N=5643)	1.27 (1.13, 1.42)	1.0 (Reference)	1.36 (1.10, 1.68)	1.65 (1.30, 2.09)	<0.001	

†ORs were adjusted for Age (years), Gender (Male, Female), Residence (Urban, Rural), Marital status (Live without spouse, Live with spouse), Education level (Illiterate, Elementary school, Middle school, High school or above), Annual household income (<10000, 10000-20000, >20000), BMI (Normal, Thin, Overweight), Smoking status (Never, Ever, Current), Drinking status (Never, Abstainer, Current), Participating in social activity (Inactive, Active), Hypertension (No, Yes), Diabetes (No, Yes), and Province (Categorized by name of each province).

Table S8 Stratified and interaction analyses of between living environmental quality and risk of arthritis in longitudinal analysis.

Characteristics	Living Environmental Quality, HR (95% CI) ‡				*P*-trend	*P*-interaction
	Continuous variable	Suitable (0-1)	Moderate (2-3)	Unfavorable (4-6)		
Gender						0.668
Men (N=5689)	1.16 (0.96, 1.39)	1.0 (Reference)	1.29 (0.91, 1.82)	1.40 (0.95, 2.07)	0.115	
Women (N=5552)	1.15 (0.99, 1.33)	1.0 (Reference)	1.25 (0.94, 1.65)	1.36 (0.99, 1.86)	0.076	
Residence						0.674
Urban (N=2925)	1.12 (0.83, 1.52)	1.0 (Reference)	1.04 (0.69, 1.59)	1.35 (0.70, 2.58)	0.454	
Rural (N=8315)	1.17 (1.03, 1.33)	1.0 (Reference)	1.35 (1.03, 1.78)	1.47 (1.10, 1.97)	0.019	
Marital status						0.347
Live without spouse (N=2153)	1.34 (0.98, 1.83)	1.0 (Reference)	1.13 (0.62, 2.08)	1.67 (0.87, 3.22)	0.065	
Live with spouse (N=9089)	1.12 (0.99, 1.27)	1.0 (Reference)	1.28 (1.02, 1.62)	1.32 (1.01, 1.71)	0.080	
Participating in social activity						0.665
Inactive (N=5014)	1.10 (0.94, 1.30)	1.0 (Reference)	1.24 (0.91, 1.68)	1.28 (0.91, 1.80)	0.233	
Active (N=5259)	1.18 (0.99, 1.40)	1.0 (Reference)	1.31 (0.96, 1.78)	1.44 (1.01, 2.05)	0.058	
Hypertension						0.778
No (N=7188)	1.13 (0.98, 1.31)	1.0 (Reference)	1.42 (1.09, 1.86)	1.41 (1.04, 1.91)	0.084	
Yes (N=4040)	1.16 (0.95, 1.42)	1.0 (Reference)	1.01 (0.70, 1.47)	1.29 (0.86, 1.95)	0.137	
Diabetes						0.172
No (N=9784)	1.10 (0.97, 1.24)	1.0 (Reference)	1.28 (1.01, 1.61)	1.28 (0.98, 1.66)	0.142	
Yes (N=1434)	1.47 (1.07, 2.02)	1.0 (Reference)	1.08 (0.57, 2.04)	1.90 (0.98, 3.69)	0.018	
Annual household income (¥)						0.781
<10000 (N=6407)	1.12 (0.95, 1.31)	1.0 (Reference)	1.14 (0.84, 1.55)	1.26 (0.90, 1.77)	0.173	
10000-20000 (N=1667)	1.19 (0.88, 1.61)	1.0 (Reference)	1.16 (0.66, 2.02)	1.40 (0.75, 2.62)	0.260	
>20000 (N=3127)	1.21 (0.98, 1.48)	1.0 (Reference)	1.53 (1.04, 2.25)	1.59 (1.03, 2.46)	0.072	
Education level						0.949
Illiterate (N=4614)	1.07 (0.90, 1.27)	1.0 (Reference)	1.01 (0.72, 1.41)	1.11 (0.77, 1.60)	0.464	
Elementary school (N=2428)	1.46 (1.14, 1.86)	1.0 (Reference)	2.42 (1.45, 4.05)	2.74 (1.57, 4.78)	0.002	
Middle school (N=2525)	1.24 (0.95, 1.60)	1.0 (Reference)	1.40 (0.89, 2.22)	1.59 (0.93, 2.72)	0.109	
High school or above (N=1657)	0.90 (0.61, 1.33)	1.0 (Reference)	0.86 (0.46, 1.59)	0.81 (0.37, 1.77)	0.588	
Body mass index (kg/m^2)						0.254
Normal (N=5104)	1.00 (0.85, 1.18)	1.0 (Reference)	1.08 (0.79, 1.47)	1.03 (0.93, 1.45)	0.987	
Thin (N=645)	0.84 (0.48, 1.45)	1.0 (Reference)	1.37 (0.43, 4.34)	0.89 (0.25, 3.20)	0.525	
Overweight (N=3953)	1.40 (1.18, 1.67)	1.0 (Reference)	1.42 (1.03, 1.95)	1.98 (1.38, 2.84)	<0.001	
Smoking status						0.205
Never (N=6677)	1.09 (0.94, 1.26)	1.0 (Reference)	1.18 (0.91, 1.53)	1.22 (0.91, 1.64)	0.238	
Ever smoker (N=1375)	1.38 (0.90, 2.11)	1.0 (Reference)	1.71 (0.77, 3.82)	2.08 (0.84, 5.19)	0.140	
Current smoker (N=3185)	1.18 (0.94, 1.49)	1.0 (Reference)	1.37 (0.87, 2.16)	1.50 (0.90, 2.50)	0.154	
Drinking status						0.534
Never (N=6563)	1.18 (1.02, 1.36)	1.0 (Reference)	1.29 (0.99, 1.70)	1.44 (1.06, 1.96)	0.025	
Abstainer (N=888)	0.86 (0.55, 1.33)	1.0 (Reference)	0.83 (0.37, 1.84)	0.73 (0.29, 1.80)	0.492	
Current drinker (N=3781)	1.18 (0.94, 1.47)	1.0 (Reference)	1.38 (0.90, 2.10)	1.47 (0.91, 2.37)	0.158	

‡HRs were adjusted for Age as time scale, Gender (Male, Female), Residence (Urban, Rural), Marital status (Live without spouse, Live with spouse), Education level (Illiterate, Elementary school, Middle school, High school or above), Annual household annual income (<10000, 10000-20000, >20000), BMI (Normal, Thin, Overweight), Smoking status (Never, Ever, Current), Drinking status (Never, Abstainer, Current), Participating in social activity (Inactive, Active), Hypertension (No, Yes), Diabetes (No, Yes), and Province (Categorized by name of each province).
